# Prognostic nomogram for estimating survival in patients with resected muscle-invasive bladder cancer receiving chemotherapy

**DOI:** 10.3389/fsurg.2023.1121184

**Published:** 2023-02-24

**Authors:** Bing Hu, Ru Chen, Guoxian Chen, Ping Zheng, Bin Fu

**Affiliations:** ^1^ Department of Urology, The First Affiliated Hospital of Nanchang University, Nanchang, China; ^2^ Department of Urology, The First Hospital of Putian City, Putian, China; ^3^ Department of Urology, Shangrao municipa0000l Hospital, Shangrao, China

**Keywords:** muscle-invasive bladder cancer, SEER database, chemotherapy, prognosis, nomograms

## Abstract

**Background:**

Chemotherapy has been proven to bring survival benefit in patients with resected muscle-invasive bladder cancer (MIBC), which is increasingly recommended. Our objective was to establish an effective model for estimating the overall survival (OS) and cancer-specific survival (CSS) in these patients.

**Methods:**

2,030 patients diagnosed with resected MIBC receiving chemotherapy were acquired from the Surveillance, Epidemiology, and End Result (SEER) database, which were randomized 7:3 into a primary set (1,421 patients) and an internal validation set (609 patients). Significant predictors for OS and CSS were identified by Cox regression models, which were then utilized to develop prognostic nomogram. The performance of the model was assessed by utilizing calibration, area under the receiver operating characteristic curve (AUC) and decision curve analysis (DCA).

**Results:**

Six independent prognostic factors, including age, race, histology, T stage, N stage and regional nodes examined, made up the nomogram. The AUCs of the primary cohort was 0.751 and 0.753 for 3- and 5- year OS and 0.751 and 0.754 for 3-and 5- year CSS, respectively. The calibration plots proved the nomograms' satisfactory discrimination. The results of DCA manifested that our models had an excellent clinical applicability. In addition, a risk stratification system was established according to the nomogram' risk score. Obvious difference was found in different groups (*P* < 0.001).

**Conclusion:**

The established prediction nomogram provides a simple-to-use tool for estimating the survival probability of resected MIBC patients treated with chemotherapy, which can assist clinicians make individualized treatment plans.

## Introduction

As the ninth most common malignancy in the world, bladder cancer (BC) is characterized by high incidence rate and high mortality (1). In 2020, there were an estimated 573,278 new cases of BC and almost 212,536 cancer deaths (2). About 25% of initially diagnosed BC were discovered to intrude into muscle tissue. Muscle invasive bladder cancer (MIBC) presents a greatly poor prognosis, whose 5-year overall survival rate is 40%–50%. Radical cystectomy (RC) is regarded as the standard treatment for MIBC. However, 50% of the patients developed metastatic bladder cancer after operation (3). Simple surgical treatment can't achieve ideal results for MIBC patients. Therefore, systemic therapy is of great significance for ameliorating the prognosis of MIBC (4). At present, according several international guidelines and research, cisplatin-based neoadjuvant chemotherapy (NAC) before RC is recommended, while adjuvant chemotherapy is recommended for patients who have not treated with NAC (4–6). Therefore, in the real world, a large proportion of resected MIBC patients are receiving chemotherapy.

In recent years, many researchers have been devoted to exploring the prognostic factors of postoperative bladder cancer. Shariat et al. constructed multivariate model for estimating the overall survival and cancer-specific survival in bladder cancer patients receiving radical cystectomy (RC) (7). And the model had been well verified externally (8). In addition, Welty et al. developed a risk-stratification tool to estimate mortality after RC based a large population cohort (9). However, few people have explored the prognostic factors of MIBC patients who receive RC and chemotherapy. Considering the poor outcomes of treatment with RC alone, it is very essential to construct a predictive model to evaluate prognostic factors in these patients.

Nomogram is a sound and extensively employed tool for evaluating the prognosis of tumors, which can integrate clinical and demographic factors to provide individualized prognostic assessment (10). In this study, we obtained clinical data from the SEER database to determine significantly prognostic factors. According these factors, we constructed a comprehensive model for accessing the overall survival (OS) and cancer-specific survival (CSS) of MIBC patients who received RC and chemotherapy.

## Methods

### Patient selection

Clinicopathological data of patients with resected MIBC receiving chemotherapy were obtained from the SEER database utilizing SEER*Stat 8.3.9 software. The inclusion criteria were as follows: (1) diagnosed with MIBC from 2004 to 2015 as the first only malignancy; (2) histological type: Transitional cell carcinoma; (3) patients treated radical cystectomy and chemotherapy (Whether adjuvant chemotherapy or neoadjuvant chemotherapy). Exclusion criteria: (1) M stage: M1 or Mx; (2) patients receiving radiotherapy; (3) the information of N stage, grade, tumor size, race, marital status and regional nodes examined unknown. Our study was approved by the Ethics Committee of the First Affiliated Hospital of Nanchang University.

### Variables defined

**Figure 1:**
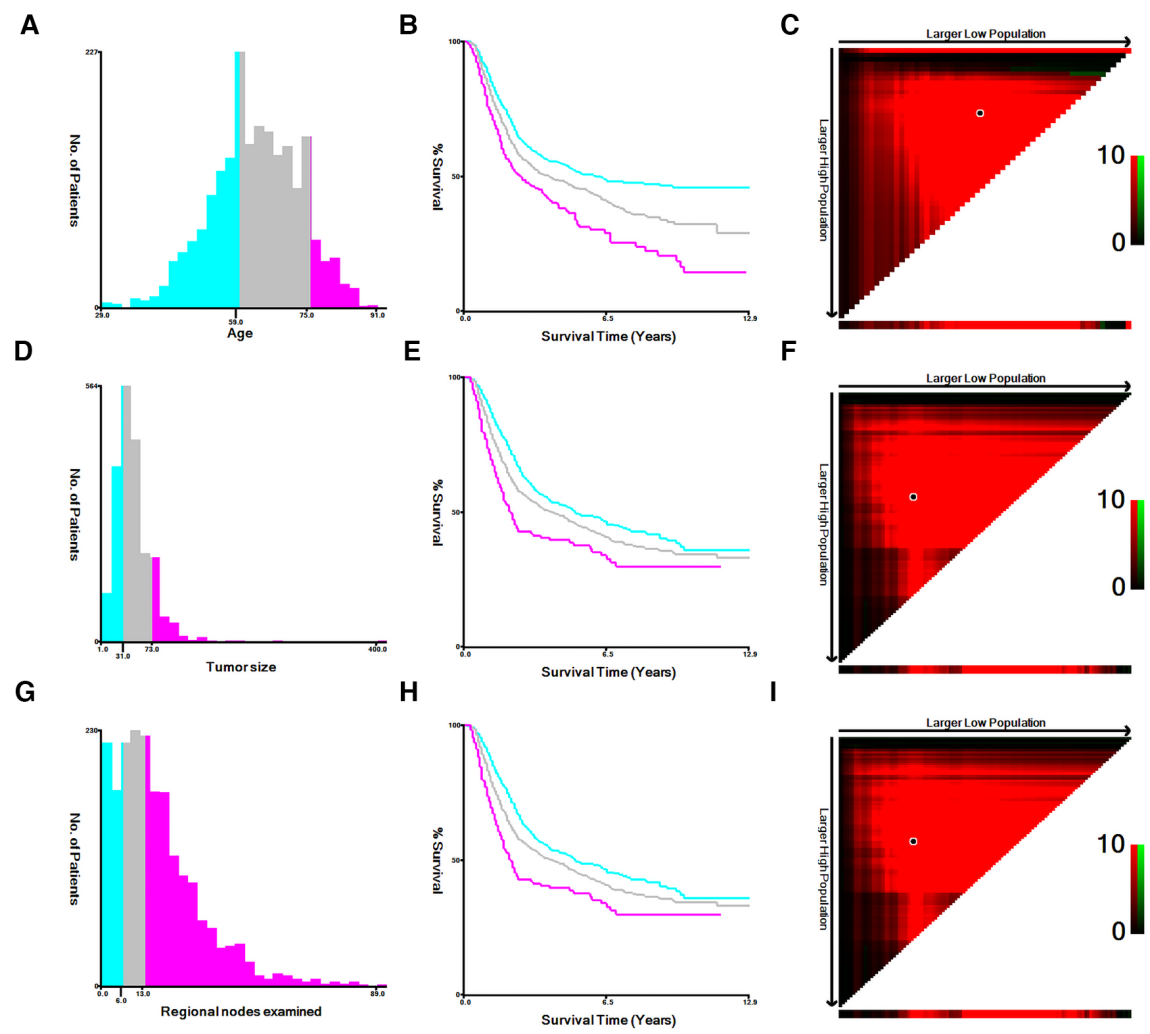
The appropriate cutoff values of age, tumor size and regional nodes examined were by X-tile analysis. (**A–C**) The appropriate cutoff values of age were 59 and 75 years. (**D–F**) The appropriate cutoff values of tumor size were 31 and 73 mm. (**G–I**) The appropriate cutoff values of regional nodes examined were 6 and 13.

The following variables included in this study were age, gender, race, marital, histology, T stage, N stage, AJCC stage, grade, regional nodes examined, summary stage, survival months and survival status. In order to obtain the optimum cutoff points of age, tumor size and regional nodes examined, X-tile software was utilized (11). Age was divided into three subgroups: <60, 60–75 and >75. Tumor size was defined as ≤30 mm, 31∼73 mm and ≥74 mm. Regional nodes examined was classified into three categories: 0∼5, 6∼13 and >13 (Figure 1). The main endpoint was OS, which was regarded as the duration between the time of diagnosis and death for any causes.

### Statistical analysis

All statistical analysis in this study were carried out by utilizing SPSS 25.0, X-tile and R software. Statistical testing was bilateral and *P* < 0.05 was considered statistically significant. First of all, the univariate and multivariate Cox regression were carried out to determine the significantly independent prognostic factors. According to these significant variables, a practical nomogram was established. The values of area under the receiver operating characteristic curve (AUC) were applied to assess the discrimination of the model. Calibration plot were utilized to estimate the difference between observed and predicted survival probabilities, and decision curve analysis (DCA) was applied to verify the model's clinical utility. In addition, according to the established nomogram risk score, we constructed a risk stratification system where we divided all patients into three categories: high-, medium- and low-risk groups. Kaplan-Meier curves and log-rank tests were applied to explore whether there were differences among different groups.

## Result

### Patient characteristics

In total, 2,030 patients diagnosed with MIBC and receiving radical cystectomy and chemotherapy were collected, including 1,421 in the primary cohort and 609 in the internal validation cohort (Supplementary Figure S1). Table 1 revealed the detailed baseline demographic and clinicopathological characteristics. The median survival time was 26.0 months (interquartile range: 1.00–155), 28.0 months (interquartile range: 1.00–155) in the primary set and internal validation set, respectively. In the SEER cohort, the 3- and 5-year OS rates were 54.3% (95%CI, 51.6% to 57.2%) and 47.2% (95%CI, 44.3% to 50.3%) in the primary set, respectively, while 56.5% (95%CI, 52.4% to 60.8%) and 44.8% (95%CI, 40.3% to 49.7%) in the internal validation set, respectively.

**Table 1:** Demographic characteristics of the primary cohort and the internal validation cohort.

Characteristics	SEER database	
	Primary cohort (%)	Internal validation cohort (%)
All	1,421	609
**Age**		
<60	473 (33.3)	207 (34.0)
60–75	770 (54.2)	336 (55.2)
>75	178 (12.5)	66 (10.8)
**Sex**		
Male	953 (67.1)	397 (65.2)
Female	468 (32.9)	212 (34.8)
**Race**		
White	1,254 (88.2)	524 (86.0)
Black	83 (5.8)	40 (6.6)
Other	84 (5.9)	45 (7.4)
**Marital status**		
Married	911 (64.1)	399 (65.5)
Unmarried	191 (13.4)	76 (12.5)
SDW	319 (22.4)	134 (22.0)
**Grade**		
I/II	28 (2.0)	12 (2.0)
III	352 (24.8)	163 (26.8)
IV	1,041 (73.3)	434 (71.3)
**Histological type**		
TCC	1,013 (71.3)	423 (69.5)
PTCC	408 (28.7)	186 (30.5)
**T stage**		
T2	576 (40.5)	238 (39.1)
T3	589 (41.4)	253 (41.5)
T4	256 (18.0)	118 (19.4)
**N stage**		
N0	890 (62.6)	345 (56.7)
N1	265 (18.6)	124 (20.4)
N2	256 (18.0)	137 (22.5)
N3	10 (0.7)	3 (0.5)
**AJCC stage**		
II	458 (32.2)	183 (30.0)
III	417 (29.3)	156 (25.6)
IV	546 (38.4)	270 (44.3)
**Tumor size**		
≤30 mm	468 (32.9)	208 (34.2)
31–73 mm	799 (56.2)	342 (56.2)
≥74 mm	154 (10.8)	59 (9.7)
**Regional nodes examined**		
0–5	243 (17.1)	86 (14.1)
6–13	406 (28.6)	178 (29.2)
>13	772 (54.3)	345 (56.7)

## Identification of prognostic factors of OS and CSS in the primary cohort

In order to obtain independent prognostic factors related to OS and CSS, we completed univariate and multivariate Cox regression analysis. According to the results of this univariate Cox regression analysis, eight factors (age, race, histology, tumor size, T stage, N stage and regional nodes examined) were associated with the main outcome OS and CSS. In multivariate Cox regression analysis, six variables (age, race, histology, T stage, N stage and regional nodes examined) were determined as independent predictors, whether OS (Table 2) or CSS (Table 3) in resected MIBC patients treated with chemotherapy.

**Table 2:** Univariable and multivariate Cox proportional hazards regression analysis for OS in the primary cohort.

	Univariate analysis			Multivariate analysis		
	OR	95% CI	*P* value	OR	95% CI	*P* value
**Age (years)**						
<60	Ref			Ref		
60–75	1.276	1.076–1.514	**0**.**005**	1.281	1.077–1.523	**0**.**005**
>75	1.743	1.381–2.202	**<0**.**001**	1.716	1.351–2.178	**<0**.**001**
**Sex**						
Male	Ref			Ref		
Female	1.208	1.035–1.411	**0**.**017**	1.068	0.910–1.252	0.421
**Marital status**						
Married	Ref		** **	-		
Unmarried	1.072	0.857–1.343	0.542	-	-	-
SDW	1.120	0.936–1.340	0.216	-	-	-
**Race**						
White	Ref			Ref		
Black	1.588	1.199–2.103	**0**.**001**	1.348	1.007–1.805	**0**.**045**
Other	0.808	0.577–1.130	0.213	0.771	0.550–1.080	0.131
**Histologic type**						
TCC	Ref			Ref		
PTCC	0.650	0.544–0.778	**<0**.**001**	0.733	0.612–0.879	**<0**.**001**
**Size**						
≤30 mm	Ref			Ref		
31∼73 mm	1.262	1.067–1.493	**0**.**006**	1.101	0.928–1.307	0.270
≥74 mm	1.560	1.214–2.004	**<0**.**001**	1.140	0.874–1.486	0.333
**Grade**						
GI/II	Ref			-		
GIII	1.143	0.687–1.902	0.607	-	-	**-**
GIV	1.038	0.630–1.708	0.885	-	-	-
**T stage**						
T2	Ref			Ref		
T3	2.270	1.890–2.726	**<0**.**001**	1.684	1.388–2.042	**<0**.**001**
T4	3.273	2.657–4.030	**<0**.**001**	2.265	1.805–2.841	**<0**.**001**
**N stage**						
N0	Ref			Ref		
N1	2.362	1.967–2.836	**<0**.**001**	2.073	1.719–2.501	**<0**.**001**
N2	2.963	2.473–3.549	**<0**.**001**	2.420	2.001–2.927	**<0**.**001**
N3	4.791	2.464–9.318	**<0**.**001**	3.236	1.648–6.355	**<0**.**001**
**Regional nodes examined**						
0–5	Ref			Ref		
6–13	0.880	0.713–1.086	0.235	0.785	0.633–0.974	**0**.**028**
>13	0.691	0.569–0.841	**<0**.**001**	0.620	0.612–0.879	**<0**.**001**

**Table 3:** Univariable and multivariate Cox proportional hazards regression analysis for CSS in the primary cohort.

	Univariate analysis			Multivariate analysis		
	OR	95% CI	*P* value	OR	95% CI	*P* value
**Age (years)**						
<60	Ref			Ref		
60–75	1.218	1.015–1.461	**0**.**034**	1.221	1.015–1.469	**0**.**034**
>75	1.662	1.294–2.137	**<0**.**001**	1.632	1.263–2.109	**<0**.**001**
**Sex**						
Male	Ref			Ref		
Female	1.282	1.086–1.512	**0**.**003**	1.132	0.955–1.341	0.153
**Marital status**						
Married	Ref		** **	-		
Unmarried	1.007	0.788–1.288	0.954	-	-	-
SDW	1.104	0.911–1.339	0.312	-	-	-
**Race**						
White	Ref			Ref		
Black	1.712	1.277–2.296	**<0**.**001**	1.421	1.048–1.926	**0**.**024**
Other	0.757	0.520–1.100	0.144	0.728	0.500–1.059	**0**.**097**
**Histologic type**						
TCC	Ref			Ref		
PTCC	0.624	0.514–0.758	**<0**.**001**	0.717	0.589–0.873	**<0**.**001**
**Size**						
≤30 mm	Ref			Ref		
31∼73 mm	1.230	1.028–1.472	**0**.**024**	1.057	0.880–1.270	0.555
≥74 mm	1.612	1.236–2.103	**<0**.**001**	1.169	0.883–1.547	0.274
**Grade**						
GI/II	Ref			-		
GIII	1.387	0.754–2.551	0.292	-	-	**-**
GIV	1.228	0.675–2.235	0.502	-	-	-
**T stage**						
T2	Ref			Ref		
T3	2.451	2.009–2.990	**<0**.**001**	1.771	1.436–2.183	**<0**.**001**
T4	3.407	2.716–4.276	**<0**.**001**	2.270	1.775–2.904	**<0**.**001**
**N stage**						
N0	Ref			Ref		
N1	2.635	2.168–3.203	**<0**.**001**	2.294	1.879–2.802	**<0**.**001**
N2	3.231	2.661–3.923	**<0**.**001**	2.588	2.110–3.173	**<0**.**001**
N3	5.592	2.869–10.897	**<0**.**001**	3.687	1.896–7.274	**<0**.**001**
**Regional nodes examined**						
0–5	Ref			Ref		
6–13	0.926	0.736–1.166	0.515	0.826	0.653–1.044	0.110
>13	0.745	0.602–0.923	**0**.**007**	0.671	0.540–0.833	**<0**.**001**

### Nomogram development

**Figure 2:**
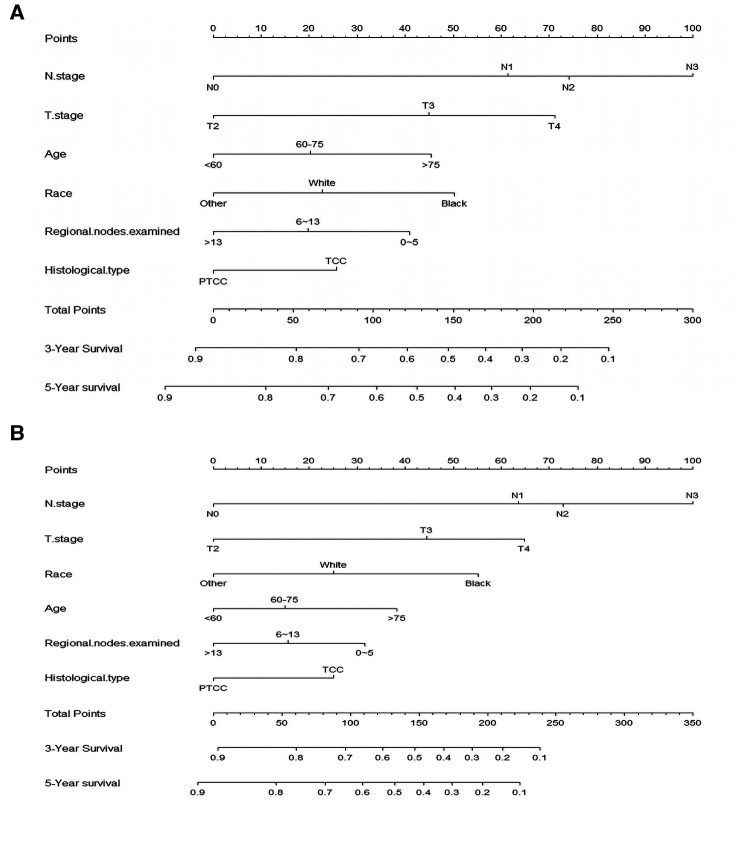
Nomogram for predicting 3-, and 5-year overall survival (**A**) and cancer -specific survival (**B**) of resected MIBC patients receiving chemotherapy.

According to the result of Cox regression models, prognostic nomogram predicting the 3-and 5-year OS and CSS in resected MIBC patients receiving chemotherapy was established (Figure 2). We can add the scores of each patient's subgroups to acquire the total score, which can be utilized to predict the survival rate of patients. Nomogram exhibited that N stage was the most important contributor to the prognostic model.

### Validation of nomogram

**Figure 3:**
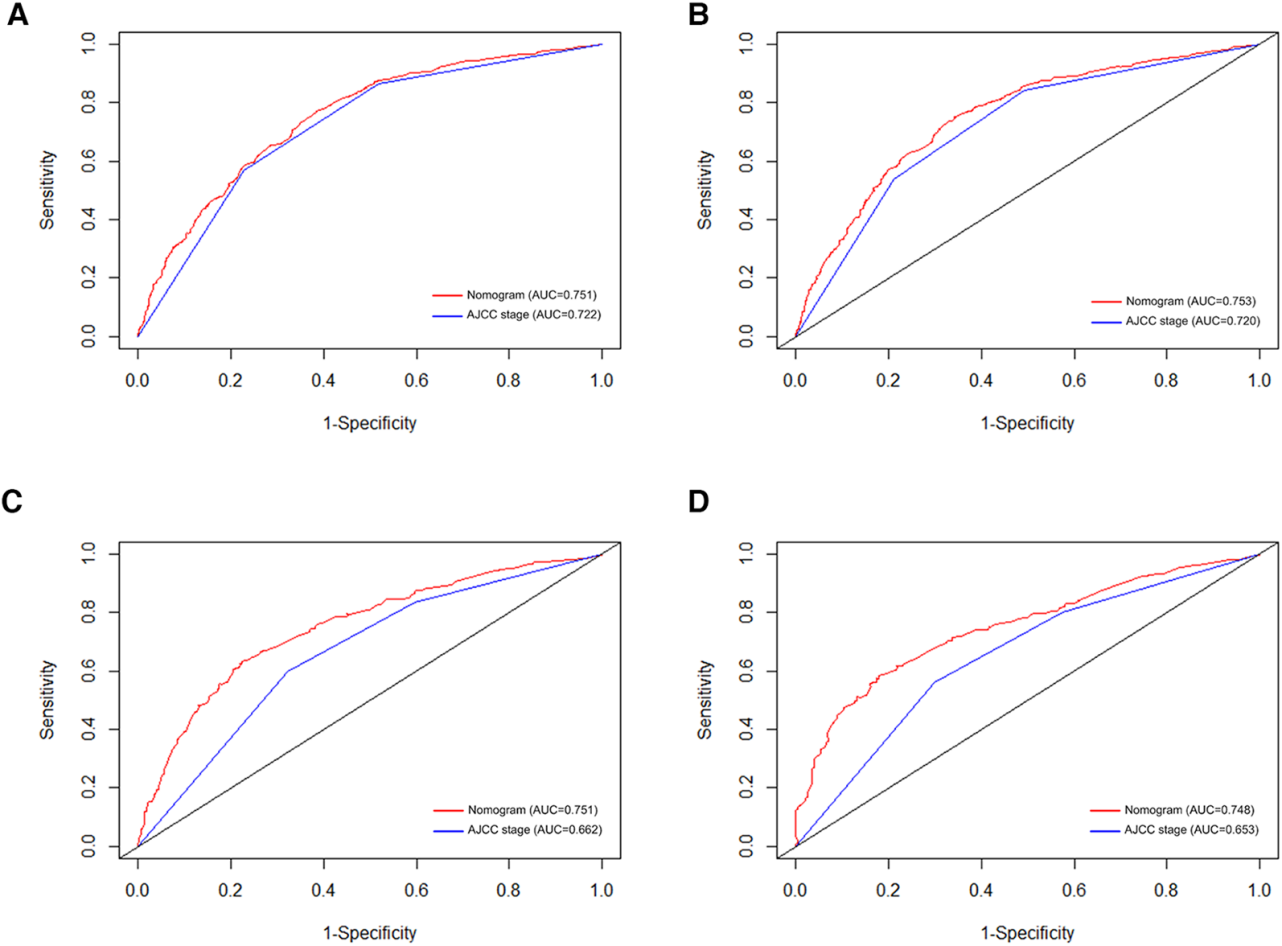
ROC curves for predicting 3-, and 5-year OS in the primary group (**A,B**), and the internal validation group (**C,D**).

**Figure 4:**
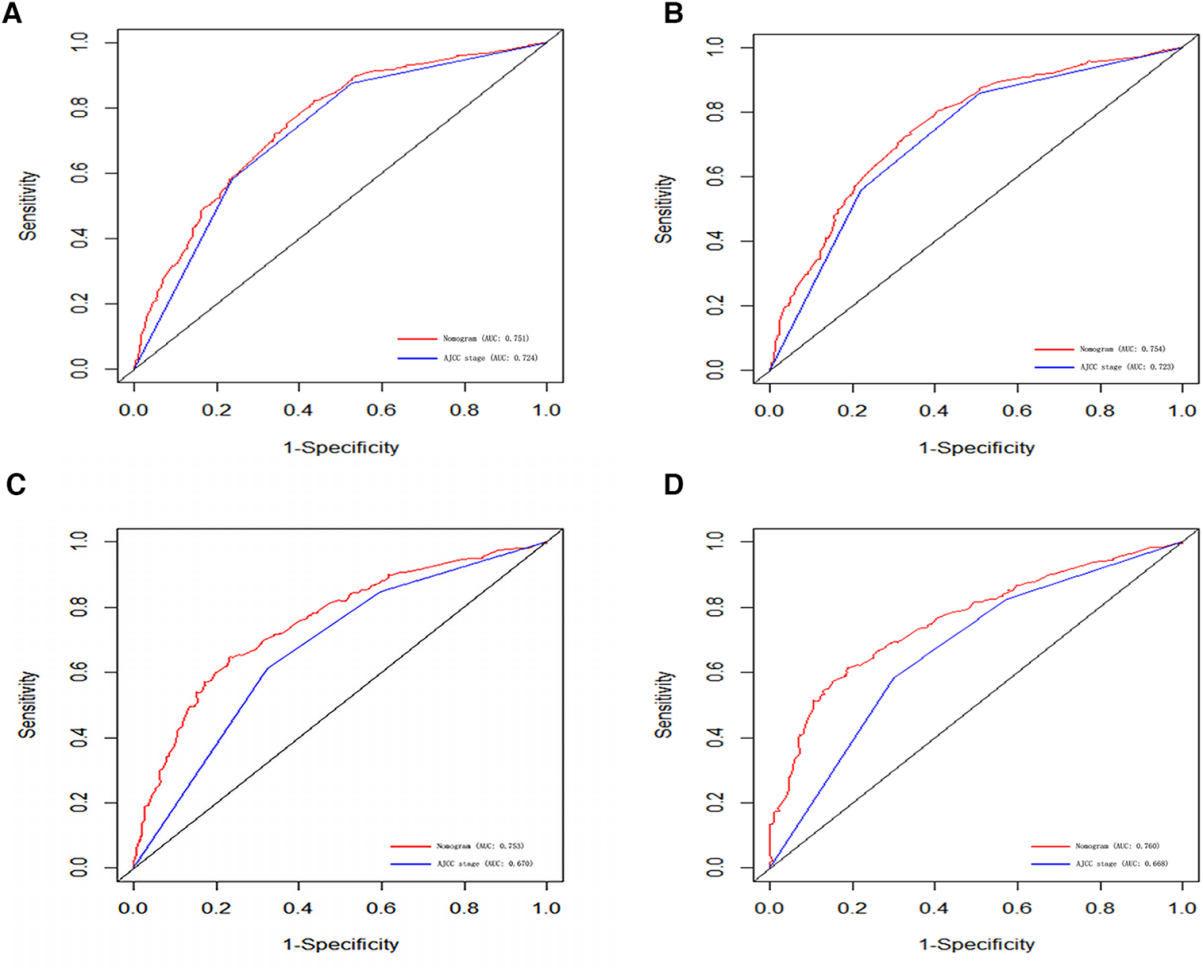
ROC curves for predicting 3-, and 5-year CSS in the primary group (**A,B**), and the internal validation group (**C,D**).

**Figure 5:**
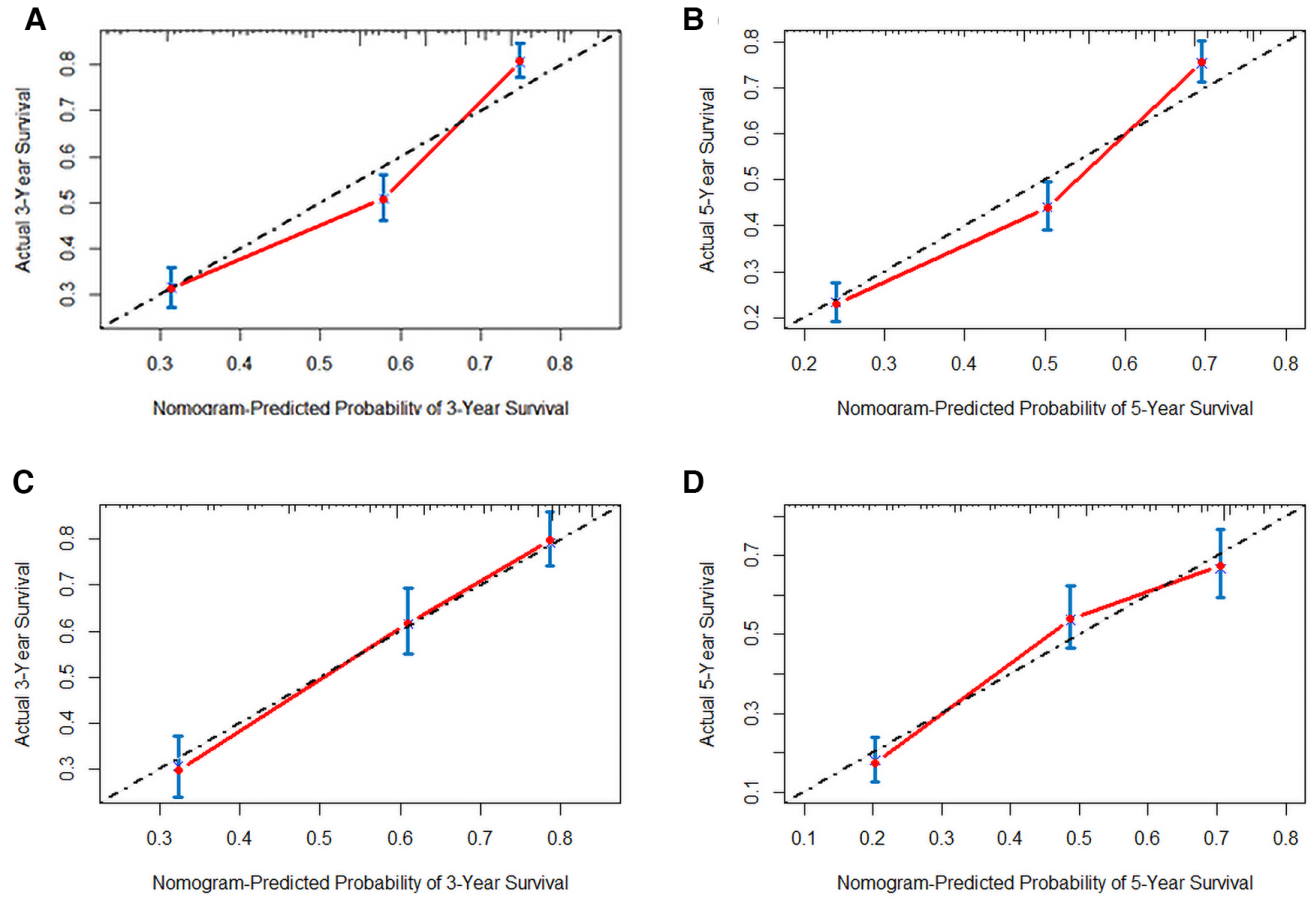
The calibration plot assessing the consistency between predicted and observed overall survival rates in 3-, and 5- years in the primary group (**A,B**) and the internal validation group (**C,D**).

**Figure 6:**
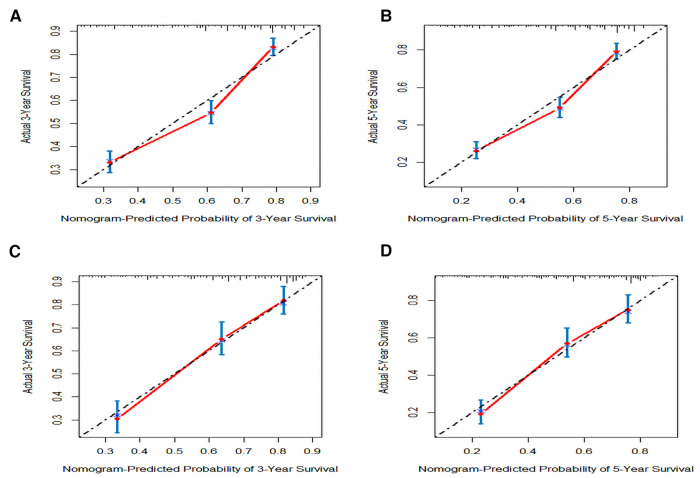
The calibration plot assessing the consistency between predicted and observed cancer-specific survival rates in 3-, and 5- years in the primary group (**A,B**) and the internal validation group (**C,D**).

**Figure 7:**
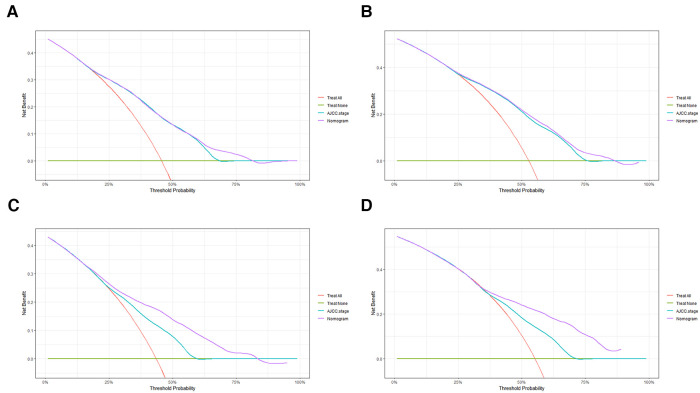
DCA to evaluate the 3-, and 5-year OS in the primary group (**A,B**) and the internal validation group (**C,D**).

**Figure 8:**
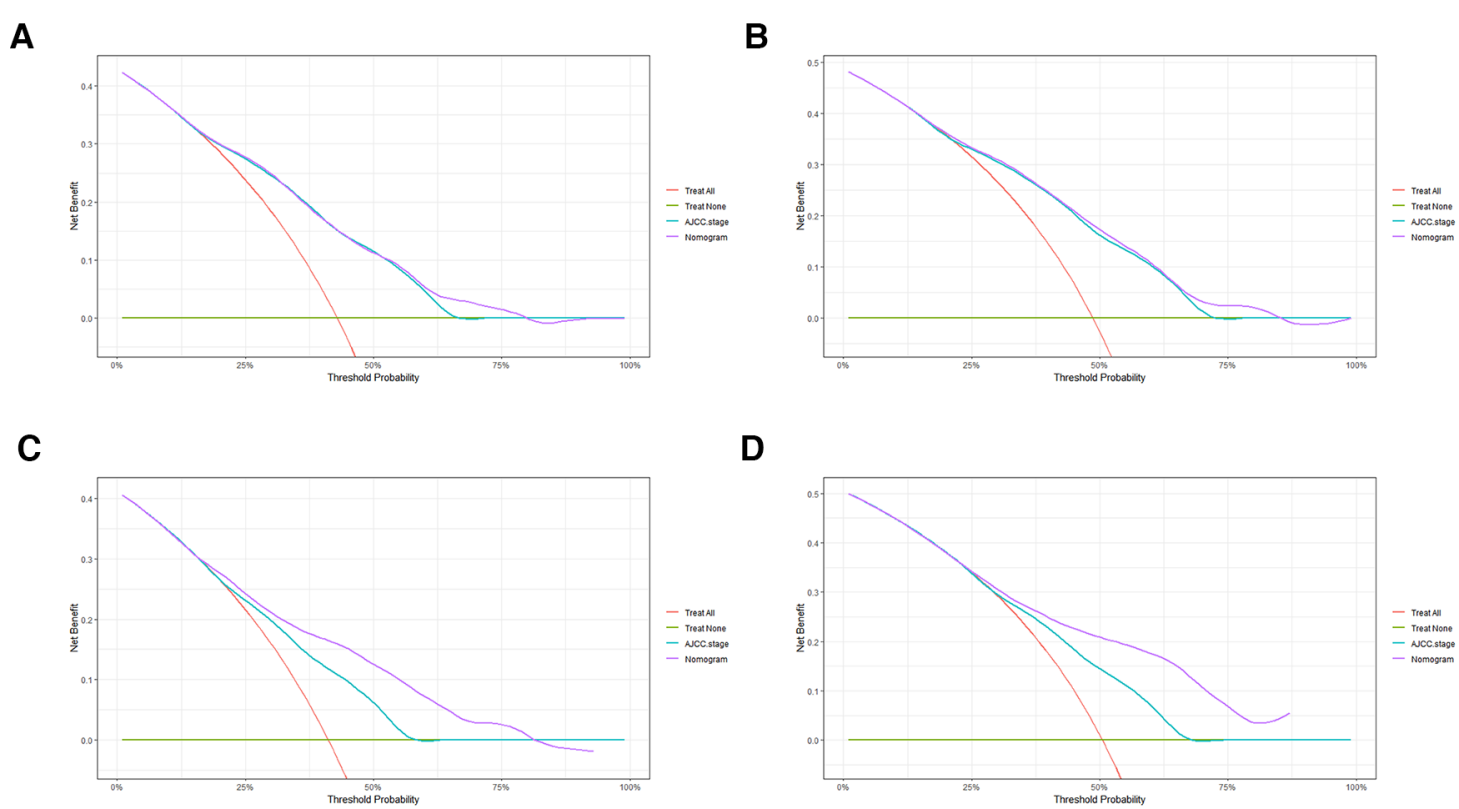
DCA to evaluate the 3-, and 5-year CSS in the primary group (**A,B**) and the internal validation group (**C,D**).

In order to further examine the perfection of our model, AJCC staging system was applied to compare with our model. In the training group and the internal validation group, the C-index of the nomogram was 0.699 (95% CI: 0.679–0.719) and 0.703 (95% CI: 0.672–0.734) for OS and 0.705 (95% CI: 0.685–0.725) and 0.708 (95% CI: 0.677–0.739) for CSS, respectively. Compared with AJCC stage, our model' C-index had a slight advantage. The AUC values of 3- and 5-years for OS (Figure 3) and CSS (Figure 4) were further calculated, whose results stayed ahead of AJCC stage. The calibration plot manifested good agreement between the actual observations and the predicted results, whether OS (Figure 5) or CSS (Figure 6). DCA also revealed that the net return of our model exceeded the broad threshold rate of AJCC stage (Figures 7, 8).

### Risk stratification

**Figure 9:**
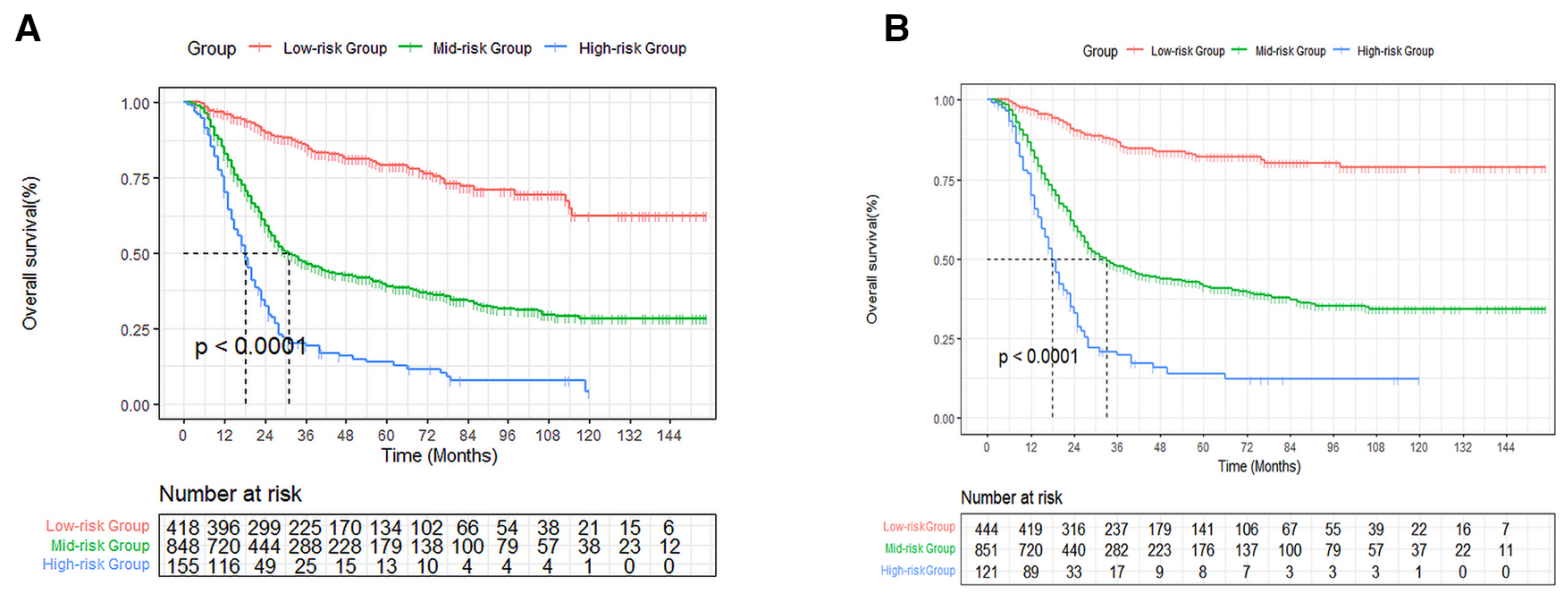
Survival curves stratified by the score calculated by the nomogram. low-risk group (score <89); medium group (score 89–201); high-risk group (score >201) for OS (**A**) and CSS (**B**).

Based on the established nomogram risk score, a risk stratification system was constructed. We utilized the X-tile software to obtain the best cutoff values of total risk score (Supplementary Figure S2). Then we divided all patients into three categories: high- (total score > 201), medium- (89 ≤ total score ≤ 201) and low-risk (total score < 89) groups. As seen in Figure 9, there were significant differences between the three risk subgroups (*P* < 0.001).

## Discussion

Muscle-invasive bladder cancer (MIBC) is usually a highly aggressive malignancy, characterized by early and most distant recurrence (12). Given the high recurrence rate and adverse results of RC alone in the treatment of MIBC, chemotherapy is essential to ameliorate the prognosis of MIBC patients (4, 13). At present, cisplatin-based neoadjuvant chemotherapy (NAC) and RC are the standard treatment for MIBC patients. International authoritative guidelines strongly recommend cisplatin-based NAC for the treatment of clinical T2–4a (cT2–4a) disease (5). In addition, in some meta-analysis and clinical research, the efficacy of adjuvant chemotherapy is affirmed for MIBC (14–16). Adjuvant chemotherapy is recommended for patients with pT3∼4 or lymph node metastasis. Considering the role of chemotherapy in the prognosis of MIBC and the increasing number of patients receiving chemotherapy, we established a practical model to access the prognosis of these patients.

Numerous previous studies have explored the effect of chemotherapy on MIBC. Lane et al. found that compared with RC alone, platinum NAC + RC treatment could bring the overall survival benefit but no CSS benefit in patients with persistent MIBC (17). Macoled et al. explored the trends and appropriateness of perioperative chemotherapy. They concluded that patients with lower complications, women, married status and lower disease stage were more likely to receive chemotherapy (18). In 2021, Maria et al developed a model predicting cancer-specific mortality in MIBC patients who received RC and NAC from an international consortium. Their established model consisted of lymph node metastasis, positive surgical margins and pathological stage (19). Compared with their study, our study was based on a larger population cohort. The target population of our study was not only patients receiving neoadjuvant chemotherapy, but also receiving adjuvant chemotherapy. In addition, we explored significantly prognostic variables such as age, number of lymph nodes removed, and pathological type, which are easily available in real world. Finally, the model we created was no less perfect than their nomogram, and had been well internally verified. We further created a risk stratification system according to the nomogram score, which helps clinicians identify high-risk groups in time for individualized treatment.

In our large-scale population study, age, race, histological type, regional nodes examined, N stage and T stage were identified as independent prognostic factors of OS. Several retrospective analyses had shown that whether in recurrence rate or survival rate, patients with positive lymph nodes were much higher than those without positive lymph nodes (20, 21). The 5-year overall survival rate and cancer-specific survival rate of lymph node positive patients were 25%∼35% and 31%, respectively (22). Consistent with their study, N stage had the greatest impact on patient survival outcomes in our model. T stage has been considered as an important prognostic factor for bladder cancer (23). The higher the stage, the more aggressive and malignant the tumor is. In addition, Fang etal explored the impact of race on the prognosis of bladder cancer. They found that the prognosis of black bladder cancer patients was worse than that of other races, which was consistent with our finding (24). The number of lymph node dissections has been shown to be an independent prognostic factor for many cancers (25, 26). Perera etal found increased lymph node harvest could provide oncological benefits in bladder patients (27). Similar to their study, we found that the increasing number of lymph nodes examined was related to a better prognosis.

In addition, we also compared our model with the AJCC TNM classification. The values of C-index and AUC of our model were higher than AJCC stage whether in training cohort or internal validation cohort. This showed that our model had excellent discrimination ability and accuracy. In DCA analysis, the net income of our model was also higher, indicating that our prognostic model had perfect clinical application value. A risk stratification system was also established based on the risk score of the nomogram, which may help clinicians identify high-risk groups for individualized treatment.

Some limitations existed in our study. First of all, this is a retrospective study, whose result may be affected by selection bias. Secondly, SEER database can't provide specific information about chemotherapy. the specific scheme and course of chemotherapy are lacking. In addition, we can't distinguish between adjuvant chemotherapy and neoadjuvant chemotherapy. Thirdly, some potential prognostic factors, such as surgical approach, any type of sexual sparing surgery, time from diagnosis to treatment and surgical margin status, were not included in our study which may have introduced non-negligible statistical bias when exploring survival outcome. Finally, although we have internally verified the established model, multicenter prospective studies are still required to further affirm the clinical effectiveness of the model in the future.

## Conclusion

In short, a nomogram of resected MIBC patients receiving chemotherapy was established based on six significant prognostic factors identified by Cox regression model, which can assist clinicians to estimate the 3-and 5-survival probabilities and play an important part in risk stratification and treatment decisions.

## Data Availability

The original contributions presented in the study are included in the article/**Supplementary Material**, further inquiries can be directed to the corresponding author/s.
