# Supplementary figures and images for: Prognostic nomogram for estimating survival in patients with resected muscle-invasive bladder cancer receiving chemotherapy
Source: Front Surg. 2023 Feb 24;10:1121184. doi: 10.3389/fsurg.2023.1121184 (PMC9998492; doi:10.3389/fsurg.2023.1121184)

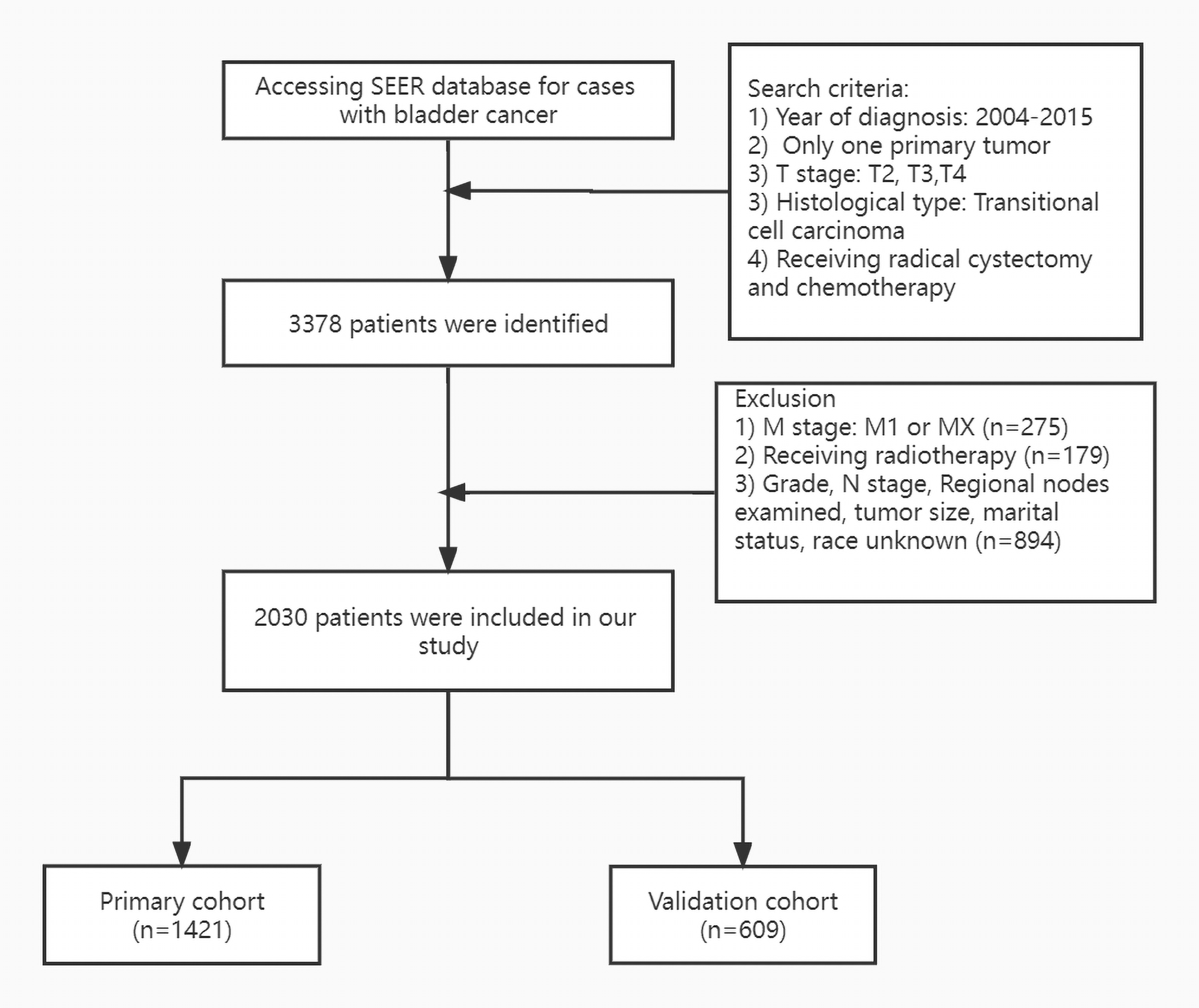

Supplement: Supplementary file 1 [file Image1.tif]

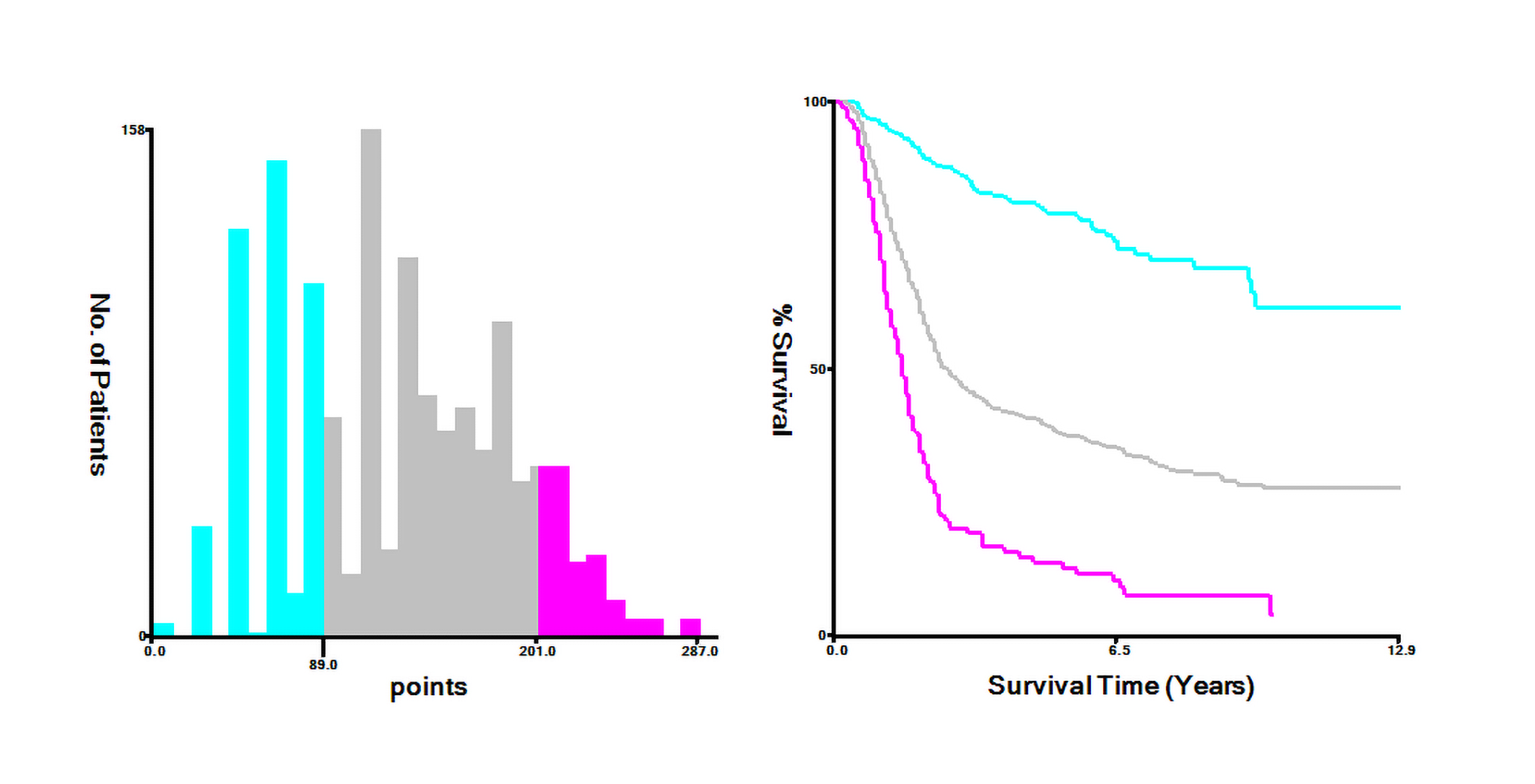

Supplement: Supplementary file 2 [file Image2.tif]
